# The complete chloroplast genome of *Styrax japonicus*

**DOI:** 10.1080/23802359.2019.1676175

**Published:** 2019-12-09

**Authors:** Zheng-Zhao Xu, Cui-Ping Zhang, Xin-Qiang Jiang, Xiao Guo, Wen-Qing Li, Qing-Hua Liu, Kui-Ling Wang, Li Wei

**Affiliations:** a College of Landscape Architecture and Forestry, Qingdao Agricultural University, Qingdao, China;; b Shandong Provincial Center of Forest Tree Germplasm Resources, Jinan, China

**Keywords:** *Styrax japonicus*, chloroplast genome, phylogenetic relationships

## Abstract

*Styrax japonicus* is a shrub with high economic values. Here, complete chloroplast (cp) genomes were reported using high-throughput Illumina sequencing. The size of the *S. japonicus* chloroplast genome is 157,940 bp long, with an average AT content of 63.05%, containing a pair of inverted repeats of 24,047 bp, separated by a large single copy and a small single copy region of 87,562 bp and 22,284 bp, respectively. It contains 125 genes, including79 protein-coding genes, 37 transfer RNA genes, and eight ribosomal RNA genes. A maximum-likelihood phylogenetic tree supported the fact that the chloroplast genome of *S. japonicus* is closely related to that of *Symplocos paniculate.*

The *Styrax japonicus* is a deciduous species within family *Styracaceae*, it is distributed from the Qin ling Mountains to the south of the Yellow River in China. The species has a broad altitude distribution range—from 400 to1804 masl (meters above sea level). The species also possesses high commercial, nutritional, and therapeutic values, and has long been used conventionally by many communities for treating oral and dental diseases and respiratory ailments. Despite the economic and medicinal significance, many of the wild *S. japonicus* populations have been seriously impacted by anthropogenic disturbances. An understanding of the genetic variability of *S. japonicus* is urgently needed to formulate appropriate conservation strategies. In this study, we assembled and characterised the complete cp genome of *S. japonicus* using next generation sequencing (Du et al. 2015). The annotated genomic sequence has been submitted to GenBank with the accession number PRJNA516732

Fresh young leaves of *S. japonicus* were collected from Lao Mountain reserve in Qingdao of Shandong province, China (36°10′N, 120°37′E). The voucher specimen was deposited in Qingdao Agricultural University (accession number: 20180510SJ01). Total genomic DNA was extracted following (Li et al. 2013). The extracted DNA was randomly fragmented to construct paired-end libraries following an Illumina preparation manual (San Diego, CA, USA).

Approximately 7.81 GB of clean data were yielded. The paired-end reads were qualitatively assessed and assembled using GetOrganelle (Jin et al. 2018) and SOAP denovo software (Luo et al. 2012). The circular chloroplast genome map was drawn using the OGDRAW programme (Marc et al. 2013).

The complete chloroplast DNA of *S. japonicus* is 157,940 bp in length. It contains a large single copy (LSC) region of 87,562 bp and a small single copy (SSC) region of 22,284 bp-separated by a pair of inverted repeats (IR) regions (24,047 bp). The overall GC content of the complete chloroplast genome is 36.95%, and GC contents of the LSC, SSC, and IR regions are 34.79%, 31.68%, and 43.32%, respectively.

A total of 124 functional genes were annotated, including 79 protein-coding genes, 37 transfer RNA, and eight ribosomal RNA. The majority of the gene species occur in a single copy and eighteen genes express two copies, including seven PCGs, nine tRNA genes.

All of the cp genome sequences were aligned using MAFFT (Kazutaka and Standley 2013). Phylogenetic analysis was constructed based on the 22 alignment sequences chloroplast genomes by maximum-likelihood (ML) analysis by RAxML based on conducted adopting Kimura 2-parameter model with 1000 bootstrap replicates (Alexandros et al. 2017). *Diospyros kaki* were used as the outgroups. As shown in the ML phylogenetic tree (Figure 1), the results based on chloroplast genome are in agreement with previous morphological and molecular analyses. The resulting tree showed that *S. japonicus* was most closely related to *S. grandiflorus* with 100% bootstrap support. The genus *Symplocos* formed a monophyletic clade and that *Symplocos paniculata* is closely related to *Styrax*. These results will lay a basis for the study of phylogeny, phylogeography, and population genetic diversity of *S. japonicus.*

**Figure 1.**
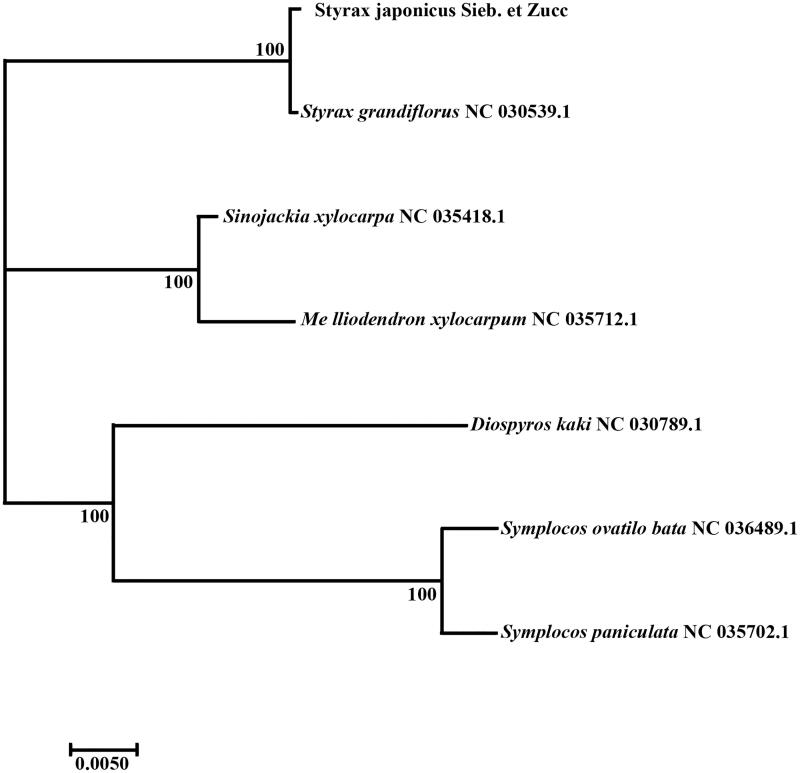
Phylogenetic tree reconstruction of seven samples using maximum-likelihood based on complete chloroplast genome. Accession number: *Styrax grandiflorus* NC 030539; *Sinojackia xylocarpa* NC 035418; *Melliodendron xylocarpum* NC 035712; *Diospyros kaki* NC 030789; *Symplocos ovatilo bata* NC 036489; *symplocos paniculata* NC 035702.
